# An unusual presentation of metastatic malignant melanoma causing jejuno-jejunal intussusception: a case report

**DOI:** 10.1186/s13256-018-1887-5

**Published:** 2018-11-13

**Authors:** Mihajlo Đokić, David Badovinac, Miha Petrič, Blaž Trotovšek

**Affiliations:** Department of Abdominal Surgery, University Medical Centre Ljubljana, Zaloška c. 7, 1000 Ljubljana, Slovenia

**Keywords:** Jejuno-jejunal intussusception, Malignant melanoma, Gastrointestinal metastasis, Metastasectomy, Emergency surgery

## Abstract

**Background:**

Small bowel intussusception in adults is rarely encountered. In most cases small bowel intussusception is caused by benign neoplastic lesions, but metastasis of cutaneous malignant melanoma causing small bowel intussusception is rare. We present such a case of jejuno-jejunal intussusception with an intraluminal metastatic lesion acting as a lead point.

**Case presentation:**

We present a case of a 71-year-old Caucasian man who presented with small bowel obstruction. His medical history revealed that he had had a cutaneous malignant melanoma excised 7 years earlier and underwent total laryngectomy due to a metastasis 6 years later. The disease was classified as stage IV and he was receiving immunotherapy. An emergency abdominal computed tomography scan demonstrated small bowel obstruction, most probably caused by an intraluminal lesion. An emergency laparotomy revealed an intraluminal metastatic lesion causing jejuno-jejunal intussusception. Metastasectomy of the lesion was performed and 13 days later he was discharged.

**Conclusions:**

Jejuno-jejunal intussusception with a malignant melanoma metastasis acting as a lead point is very rare. With the gastrointestinal tract being a common location of distal metastases, a medical history of malignant melanoma treatment in cases of small bowel obstruction should raise a suspicion of possible metastatic disease. A computed tomography scan is the diagnostic modality of choice and surgery still remains the standard of care.

## Background

Small bowel intussusception in adults is very uncommon. It accounts for only 1% of cases of bowel obstruction in adults; only 5% of all intussusceptions are found in adults [1, 2]. It is usually caused by benign neoplastic lesions, with gastrointestinal metastasis of cutaneous malignant melanoma being the cause in less than 15% of cases [3, 4]. A review of the literature offers numerous cases of different kinds of bowel obstruction due to malignant melanoma but a limited number of such cases leading to jejuno-jejunal intussusception, consequently making it interesting and important to recognize on time.

## Case presentation

A 71-year-old Caucasian man presented to our emergency room with vomiting and abdominal pain. He had been experiencing constipation and abdominal discomfort for a few weeks and had heard borborygmi in his intestine. He had noticed an occasional black stool during defecation. Due to persistent normocytic anemia, with hemoglobin levels below 100 g/L, he had had a gastroscopy, which revealed no abnormal conditions. He was also scheduled for a colonoscopy.

He had a history of arterial hypertension. Seven years earlier, he had malignant melanoma excised from his abdominal skin. Left-sided axillary lymphadenectomy was performed later due to positive sentinel node. For 6 years his clinical condition was stable. Then, a metastasis on his vocal cord and in his sternum was found, along with a suspicious lesion in his left breast. He underwent total laryngectomy and started receiving immunotherapy with vemurafenib and cobimetinib. Due to adverse side effects, including vomiting, weight loss, and phototoxicity, his therapy was adjusted to reduced dosages, which he was still receiving at the time of our encounter. A head, neck, and chest computed tomography (CT) scan performed for follow-up in another institution 1 month before admission to our department, demonstrated a stable disease.

**Fig. 1:**
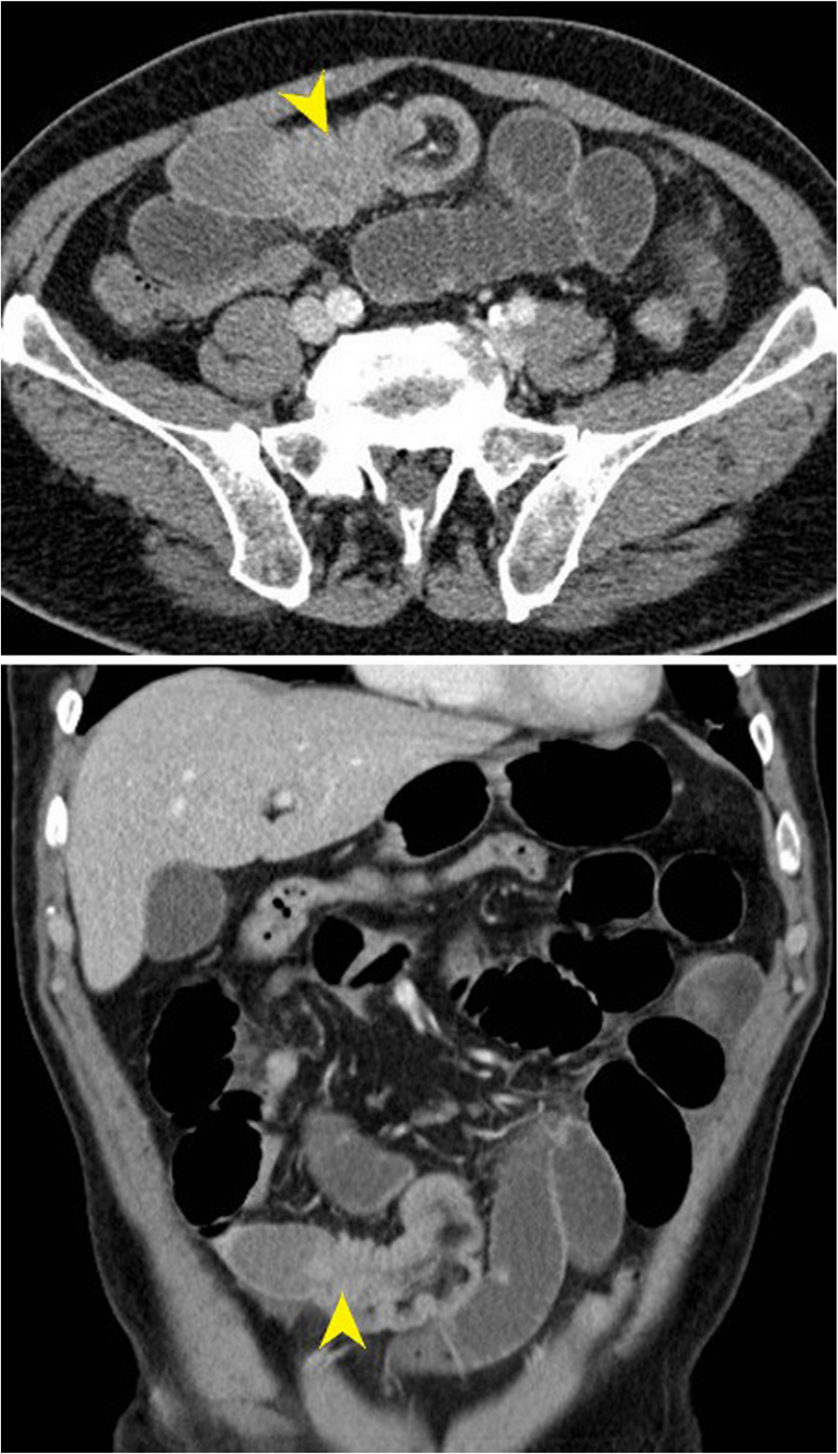
Emergency computed tomography scan demonstrating small bowel intussusception with a suspected intraluminal lesion as a lead point (*arrows*)

In our emergency room he was stable. His abdomen was distended and diffusely tender on palpation, but without any signs of peritoneal irritation. An absence of bowel sounds was discovered on auscultation. An emergency CT scan was performed, demonstrating a 10 cm long segment of small bowel intussusception (Fig. 1). The leading cause of intussusception was unclear, but the possibility of a Meckel’s diverticulum or a metastatic lesion was discussed.

**Fig. 2:**
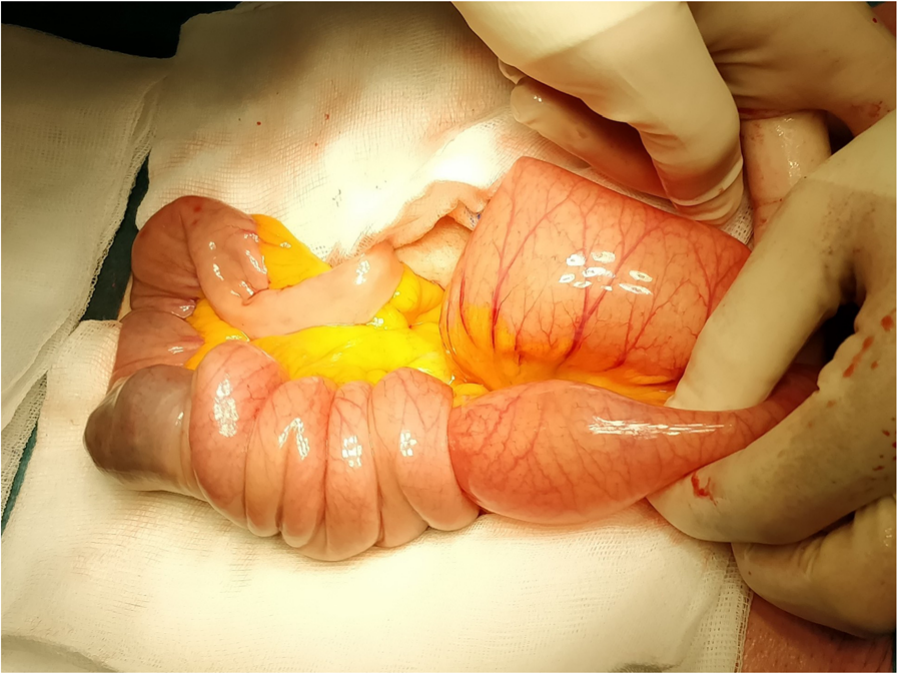
Jejuno-jejunal intussusception found at exploratory laparotomy

**Fig. 3:**
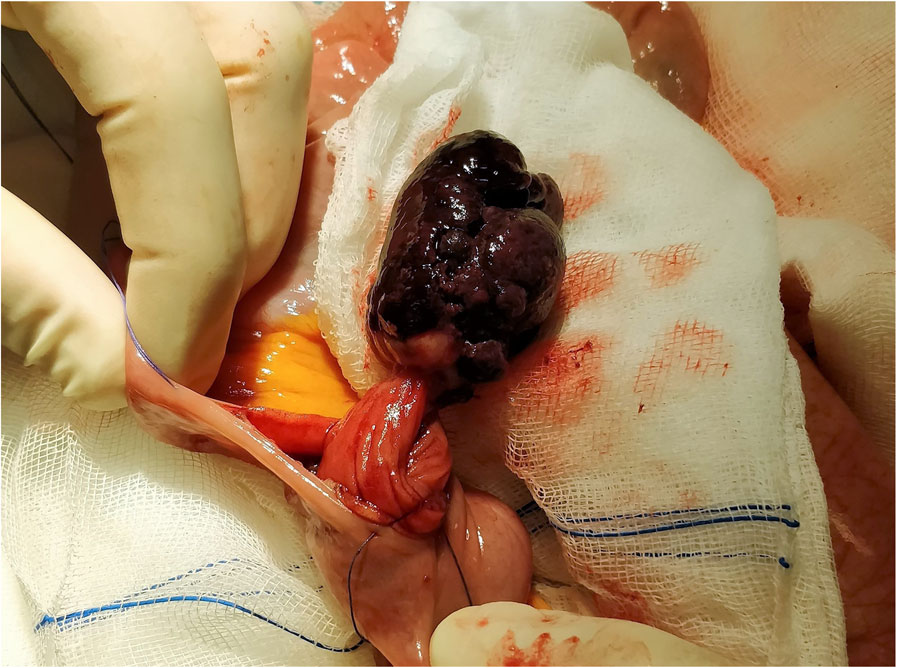
Intraluminal metastatic lesion acting as a lead point

After conservative measures and a nasogastric tube and intravenously administered fluids, he was taken to the operative theatre where an explorative laparotomy was done. His proximal small bowel was immensely distended, yet bowel motility was preserved and blood perfusion was good. Approximately 100 cm distally from the ligament of Treitz a jejuno-jejunal intussusception was found to be causing obstruction (Fig. 2). At that point an intraluminal tumor was palpable. No other abnormal conditions or suspicious lesions were found in his abdomen. First, intussusception was manually resolved. A small enterotomy at the level of the tumor revealed a pedunculated formation, measuring 5 cm in diameter (Fig. 3). Excision of the tumor along with the adherent mucosa was performed. The enterotomy was eventually closed with interrupted reabsorbable sutures. After the procedure he was admitted to our intensive care unit and a few days later to a normal hospital ward. Further hospital stay was uneventful and 13 days after admission he was discharged.

Histology of the tumor confirmed it to be a metastasis of malignant melanoma: S100, MelanA, and human melanoma black-45 (HMB-45), all positive. R0 resection was achieved. Further follow-up visits were scheduled with our patient’s treating oncologist at another institution. At the last visit, his clinical condition was stable and he resumed immunotherapy.

## Discussion

Malignant melanoma has a high metastatic potential. The gastrointestinal tract (GI) is the third most common site of distant metastases; among these, the small intestine is most commonly affected [5]. The small intestine, rectum, colon, stomach, and even the duodenum may be affected [6, 7]. Only 5–6% of patients with malignant melanoma have diagnosed GI metastases due to different clinical complications [5, 8]. However, up to 60% of patients have GI metastases that are never diagnosed during their lifetime [9]. Despite this relatively high number, metastatic melanoma causing small bowel obstruction is relatively rare.

Among all causes of bowel obstruction in adults, intussusception accounts for only 1% [1, 2]. It is much more common in childhood, whereas only around 5% of all intussusceptions occur in adulthood [2]. In children, 90% of cases are idiopathic. By contrast, 90% of adult cases have an underlying cause, usually neoplastic [3]. Of these cases, 85% are benign tumors, the rest are malignant, mostly metastatic. In this category, metastases of malignant melanoma are the most common [4].

Reported cases of jejuno-jejunal intussusception due to malignant melanoma metastases acting as a lead point are scarce. The clinical presentation of such cases is often unspecific, with abdominal pain, nausea, and vomiting being the most common symptoms. Weight loss, melena, or clinically relevant anemia are rarely seen in adults [1, 2]. However, our patient experienced such difficulties. He had had a persistent anemia due to occult intestinal hemorrhage, the cause of which had not been diagnosed prior to the operation. Other clinical symptoms he experienced were also unspecific and gradually progressed over a few weeks.

An emergency CT scan was performed at admission of our patient. An abdominal ultrasound demonstrating a donut sign can be helpful in diagnosing an intussusception due to intraluminal malignancy, but CT remains the modality of choice [10, 11]. CT has accuracy close to 80%, but it takes an experienced radiologist to recognize an intraluminal lesion acting as a lead point [12, 13]. Our radiologist managed to raise suspicion of such a cause. However, pointing out a possible reason for intussusception in such an acute setting should not change our decision to perform emergency laparotomy.

Surgery remains the standard of care of small bowel intussusception with a malignancy acting as a lead point. Usually the operation is urgent and lifesaving, considering that most of the cases are discovered only after symptoms of obstruction become obvious. Elective surgery is considered only in selected cases [14]. Whether reduction of intussusception should be performed prior to resection is unclear and there are no clear guidelines on the matter [1, 15, 16]. In selected cases, when the lead point is suspected to be benign or if safe reduction is possible, it can be performed before resection. Yet, most authors still prefer en bloc resection without preceding reduction [1, 11, 16]. In our patient, reduction of intussusception was performed. The gut was completely viable and reduction was easily achieved before complete excision of the metastasis was executed.

Malignant melanoma with distal metastases is considered a stage IV disease and such patients are subject to systemic therapy. There is no clear consensus on the optimal surgical approach. Although most authors propose resection of the affected small bowel segment, metastasectomy in stage IV disease is also adequate [17]. The primary goal remains R0 resection. Patients undergoing complete metastasectomy of GI lesions reach median overall survival of 64 months [6]. In our case, our patient also had a bone metastasis in the sternum and a lesion in his breast. Thus, the operation performed was, due to acute bowel obstruction, in fact lifesaving, but also palliative. We believe extended en bloc resection would pose a higher risk of postoperative complications in our patient receiving immunotherapy than simple enterotomy and metastasectomy which was performed.

The average life span of stage IV malignant melanoma is 2 months to 15 years after diagnosis [18]. The time from the excision of the primary cutaneous tumor to diagnosing GI metastases is also very variable. On average, it takes from 3 to 6 years for GI metastases to occur, but in some cases they can be found even more than 10 years later [10, 12, 19]. Considering the symptoms our patient had been experiencing before complete obstruction of the bowel occurred, it took around 7 years for GI metastases to arise, placing him in just above the average time expected.

## Conclusions

Malignant melanoma metastasizing to the GI tract is not uncommon, yet it rarely causes bowel obstruction. Jejuno-jejunal intussusception with a malignant melanoma metastasis acting as a lead point is seldom encountered and only a few cases have been described in the literature. However, in cases of chronic or acute small bowel obstruction a suspicion of possible metastatic disease should be raised. In such cases, a CT scan is the diagnostic modality of choice and surgery still remains the standard of care.

## Abbreviations

CT: Computed tomography
GI: Gastrointestinal tract
